# PARP inhibitors in pancreatic cancer: molecular mechanisms and clinical applications

**DOI:** 10.1186/s12943-020-01167-9

**Published:** 2020-03-02

**Authors:** Heng Zhu, Miaoyan Wei, Jin Xu, Jie Hua, Chen Liang, Qingcai Meng, Yiyin Zhang, Jiang Liu, Bo Zhang, Xianjun Yu, Si Shi

**Affiliations:** 1 Department of Pancreatic Surgery, Fudan University Shanghai Cancer Center, Shanghai, 200032 China; 2 Department of Oncology, Shanghai Medical College, Fudan University, Shanghai, 200032 China; 3 Pancreatic Cancer Institute, Fudan University; Shanghai Pancreatic Cancer Institute, Dong’An Road, Shanghai, 200032 Xuhui District China

**Keywords:** PARP inhibitor, Pancreatic cancer, BRCA, Synthetic lethality, Homologous recombination repair, Chemotherapy resistance, Biomarkers

## Abstract

Pancreatic cancer is a highly lethal disease with a poor prognosis, and existing therapies offer only limited effectiveness. Mutation gene sequencing has shown several gene associations that may account for its carcinogenesis, revealing a promising research direction. Poly (ADP-ribose) polymerase (PARP) inhibitors target tumor cells with a homologous recombination repair (HRR) deficiency based on the concept of synthetic lethality. The most prominent target gene is BRCA, in which mutations were first identified in breast cancer and ovarian cancer. PARP inhibitors can trap the PARP-1 protein at a single-stranded break/DNA lesion and disrupt its catalytic cycle, ultimately leading to replication fork progression and consequent double-strand breaks. For tumor cells with BRCA mutations, HRR loss would result in cell death. Pancreatic cancer has also been reported to have a strong relationship with BRCA gene mutations, which indicates that pancreatic cancer patients may benefit from PARP inhibitors. Several clinical trials are being conducted and have begun to yield results. For example, the POLO (Pancreatic Cancer Olaparib Ongoing) trial has demonstrated that the median progression-free survival was observably longer in the olaparib group than in the placebo group. However, PARP inhibitor resistance has partially precluded their use in clinical applications, and the major mechanism underlying this resistance is the restoration of HRR. Therefore, determining how to use PARP inhibitors in more clinical applications and how to avoid adverse effects, as well as prognosis and treatment response biomarkers, require additional research. This review elaborates on future prospects for the application of PARP inhibitors in pancreatic cancer.

## Introduction

Pancreatic cancer is a highly fatal disease with a poor prognosis. The 5-year survival rate is a mere 9%, and the incidence has steadily increased worldwide over the past 3 decades. Moreover, it is the fourth leading cause of cancer death in both males and females of all ages in the USA [1, 2]. Surgical resection is considered the only potentially curative therapy; however, only 20% of the patients diagnosed with pancreatic cancer are candidates for initial resection. Because pancreatic cancer is often asymptomatic at the early stage, the disease has typically already progressed to an advanced stage at the time of diagnosis [3, 4]. Unfortunately, even after surgical resection, most patients eventually experience recurrence [5], and they receive limited benefit from and often become resistant to chemotherapy and radiotherapy. Thus, the current state of pancreatic cancer is a grim picture, and novel drug strategies are urgently needed. It has been well acknowledged that pancreatic cancer has many different molecular subgroups with unique biological characteristics, which is partially responsible for the poor effectiveness and drug resistance observed for existing treatments [6]. Therefore, it is essential to identify the molecular mechanism of different subsets of patients with tumor genome mutations and provide individualized targeted therapies [7].

According to some comprehensive genomic analyses, four major driver genes have been identified in pancreatic cancer: KRAS, CDKN2A, TP53, and SMAD4. However, none of these genes are clinical targets in current therapeutic regimens [8–10]. Other genes associated with genetic susceptibility to pancreatic cancer [11] can be evaluated through panel-type targeted sequencing, including BRCA1 and BRCA2 [12], ATM [13], PALB2 [14], STK11 [15], the DNA mismatch repair (MMR) genes MLH1, MSH2, MSH6 and PMS2 [16], and some low-probability mutant genes, such as CHEK2, BARD1, NBN, and MUTYH/MYH [17]. Notably, the incidence of BRCA1/2 mutations fluctuates between 1 in 300 and 1 in 800 among different ethnicities [18, 19]. Pancreatic cancer is the third most common cancer related to early-onset gene mutation in breast cancer (BRCA, breast cancer susceptibility genes) as well as ovarian cancer. A family history of pancreatic cancer is an essential risk factor [20], and germline BRCA2 mutations comprise the highest proportion of known reasons for inherited pancreatic cancer [21]. Among familial pancreatic cancer patients, germline BRCA2 mutations have been observed in 5–17% [22, 23], especially in the Ashkenazi Jewish population, in which there are 10% of unselected, apparently sporadic, pancreatic cancers related to germline BRCA mutations [24]. Therefore, targeted therapy for BRCA mutations has solid genetic background support in pancreatic cancer [25].

Among the many cancer drugs that have been developed, synthetic lethality is one of the most important concepts first introduced by Bryant et al. [26] and Farmer et al. [27] as early as 2005. This concept originated from studies in drosophila model systems; a single gene/protein alteration is nonlethal, but the simultaneous inactivation of two or more genes/proteins gives rise to cellular death [28]. These tumor-specific genetic defects result in the application of targeted drugs that induce death in cancer cells while sparing normal cells [29]. In recent years, poly (ADP-ribose) polymerase (PARP) inhibitors have become the most commonly used drugs to target BRCA mutations based on this concept.

Regarding clinical trials for breast cancer, the OlampiAD phase III study (NCT02000622) demonstrates that olaparib significantly prolongs PFS in patients with metastatic breast cancer and a germline BRCA1/2 mutation compared to standard therapy [30]. The result of the EMBRCA phase III study (NCT01945775) also confirms that single-agent talazoparib provides a significant benefit over standard chemotherapy with respect to PFS among patients with advanced breast cancer and a germline BRCA1/2 mutation [31]. In the field of ovarian cancer, the SOLO1 phase III study (NCT01844986) reveals that maintenance therapy with olaparib results in a shorter PFS among women with newly diagnosed advanced ovarian cancer and a BRCA1/2 mutation, with a 70% lower risk of disease progression or death with olaparib than with placebo [32]. For prostate cancer, the TOPARP-B phase II study (NCT01682772) found that patients treated with olaparib who carried 1 or more DNA repair-related/PARPi-sensitive gene mutations had significantly improved comprehensive response rates (including objective imaging response rates, PSA response rates, and CTC conversion rates) [33]. The successful results of clinical trials for PARP inhibitors among subtypes also offer new ideas for the treatment of pancreatic cancer.

According to the NCCN Clinical Practice Guidelines in Oncology for Pancreatic Adenocarcinoma (Version3 2019.6) [34], “Germline testing is recommended for any patient with confirmed pancreatic cancer”, and “consider olaparib as maintenance treatment for patients who have a deleterious germline BRCA1/2 mutation, good performance status (defined as ECOG 0-1, with good biliary drainage and adequate nutritional intake, and ECOG 0-2 if considering gemcitabine + albumin-bound paclitaxel), metastatic disease, and no disease progression during >16 weeks of first-line, platinum-based chemotherapy.” Therefore, the use of PARP inhibitors in pancreatic cancer has broad prospects and may bring hope to this challenging disease.

This review mainly introduces the concept of synthetic lethality and homologous recombination, describes the mechanism of action of PARP inhibitors within this concept, discusses problems such as resistance, enumerates the current progress and achievements of clinical trials for PARP inhibitors in pancreatic cancer, provides examples of biomarkers for prognosis and treatment response, and summarizes the application prospects and potential problems related to the use of PARP inhibitors for pancreatic cancer.

## The concept of synthetic lethality and HRR

Preservation of the genetic code is critical for healthy cells; thus, an interrelated series of molecular pathways are used by the cell to recognize and repair DNA damage [35]. The lack of a DNA damage response will lead to the introduction of mutations that drive normal cells towards proliferation and dysfunction, sometimes leading to cancer [36].

Six primary pathways of DNA repair have been identified; four of the six repair pathways that sense single-stranded DNA breaks (SSBs) are base excision repair (BER), nucleotide excision repair (NER), mismatch repair (MMR), and trans-lesional synthesis [35, 37]. In the event that SSB repair is defective, double-stranded DNA breaks (DSBs) can form, and two other mechanisms will compensate for this deficiency. The first is homologous recombination repair (HRR), a form of repair that uses the sister chromatid as a template to restore the original DNA sequence; this mechanism is a high-fidelity system and seems to be preferred. The second is nonhomologous end-jointing (NHEJ), which is more error-prone and easily results in chromosomal aberrations as well as more subtle DNA mutations [37–39]. These two DSB repair pathways could act as compensatory mechanisms and maintain the integrity of the genome.

**Fig. 1:**
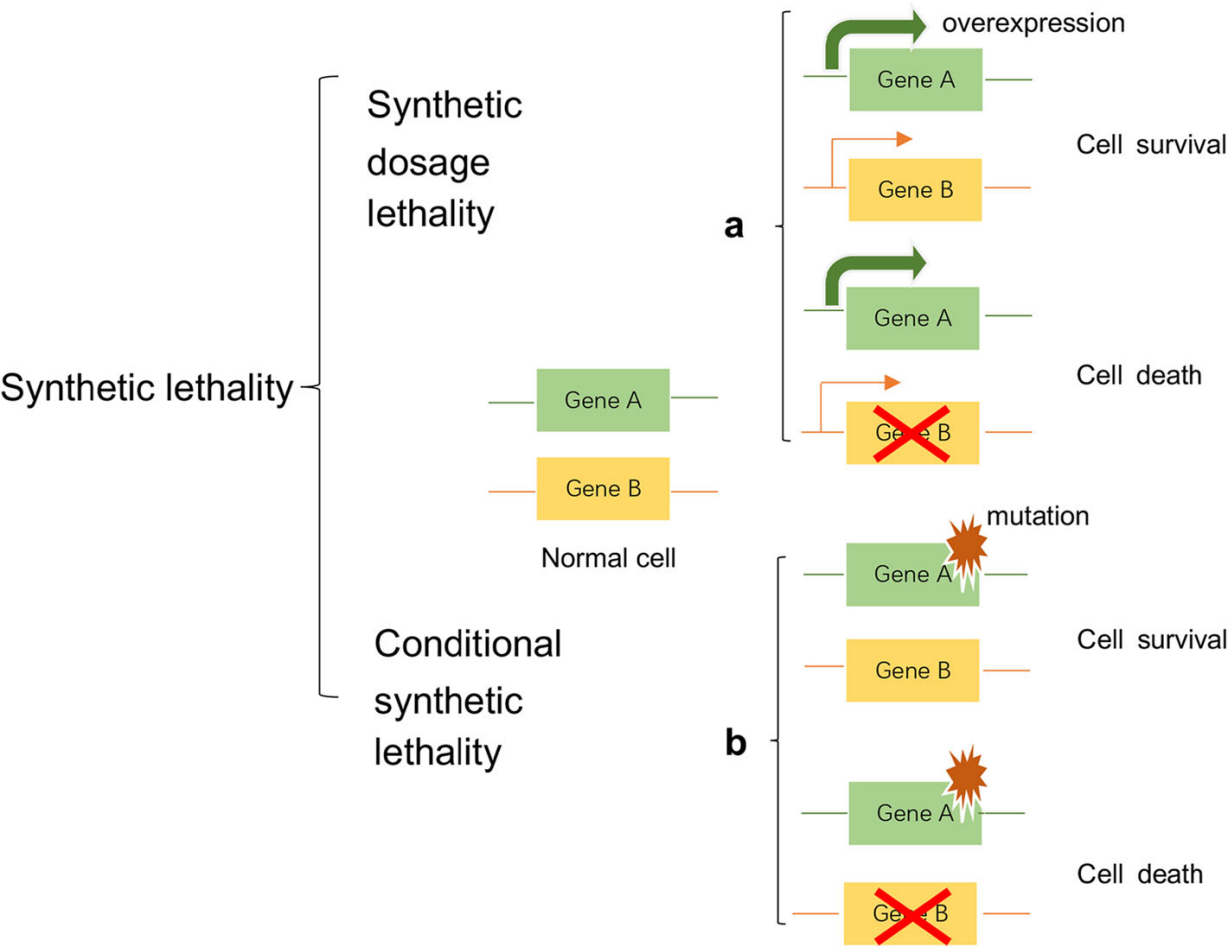
[The concept of synthetic lethality] Synthetic lethality is defined as a combination of mutations in two or more separate genes or proteins that induce cell death. For example, if a cell suffers the loss or inhibition of either gene/protein A or B alone, it remains viable, while mutation or pharmacological inhibition of an interaction partner of gene/protein **a** or **b** will result in cell death. Synthetic dosage lethality (SDL) (part **a**). Conditional synthetic lethality (part **b**)

In recent years, synthetic lethality, which has attracted great interest among geneticists and developmental biologists and is widely studied in various disease fields, has been defined as a combination of mutations in two or more separate genes or proteins that induces cell death [40, 41]. With research on biological tumor behavior and its molecular mechanisms, the concept of synthetic lethality has inspired researchers and clinicians to determine whether a synergistic lethal gene of a major mutant gene exists because many genetic mutations exist in cancer cells. Moreover, approaches based on this concept could be expanded beyond targeting loss-of-function mutations in cancer cells [42]. The concept of synthetic lethality can be divided into synthetic dosage lethality (SDL) and conditional synthetic lethality. SDL is a genetic interaction between two genes where the inhibition of gene/protein A combined with the overexpression of gene/protein B is lethal to cells (Fig. 1a) [43]. Conditional synthetic lethality depends on certain intrinsic conditions, such as genetic background, hypoxia or metabolic changes, or extrinsic conditions, such as the application of DNA-damaging drugs (Fig. 1b) [42]. Understanding and applying synthetic lethality would greatly promote the development of new targeted drugs for cancer therapy.

## Mechanisms of PARP

According to this principle, PARP inhibitors have received great attention. PARP is a nuclear enzyme, and 18 members of the PARP protein family [44] that transfer PAR or mono-ADP-ribose to themselves and/or other target proteins have been identified; among them, PARP-1 plays a major role in the total activity [29]. PARP-1 is considered a DNA nick sensor and occupies a central position in DNA SSBs, especially BER. In addition, PARP-1 plays a role in activating ATM, which is essential for HR, and inactivating DNA-dependent protein kinases, which play an important role in NHEJ [45]. PARP-1 catalyzes the movement of ADP-ribose molecules from NADC to itself and other acceptor proteins to generate PARP chains [46], which recruit DNA repair proteins, such as DNA polymerase β and DNA ligase III, and scaffolding proteins, such as X-ray cross-complementing protein 1 (XRCC1), to SSB lesions [47]. PARP-1 may also assist in HR by recruiting factors such as ATM, Mre11, and Nbs1 to DSB lesions [48]. The non-DNA bound state of PARP-1 shows a relatively disordered conformation, as “beads on a string”, and can sense DNA damage and bind to DNA lesions at SSBs via a zinc finger DNA-binding domain [49]. After binding to damaged DNA mainly through a second zinc finger domain, PARP-1 forms a homodimer and catalyzes nicotinamide adenine dinucleotide (NAD) + cleavage to nicotinamide and ADP-ribose, which are then used to synthesize branched-chain nucleic acid polymers. Poly (ADP-ribose) (PAR) covalently binds to nuclear receptor proteins. Branched polymers range in size from a few to 200 ADP-ribose units. Due to their high negative charge, covalently linked ADP-ribose polymers greatly affect the function of the target protein. Then, the helical domain of PARP-1 undergoes a conformational change that inhibits its autoinhibitory function and enhances its catalytic activity. PARP-1 recruits various DNA repair effectors, such as the molecular scaffold protein XRCC1, to the site of the lesion [47], using NAD^+^ to branch the polymers of PAR chains (poly-ADP-ribosylation, PARylation). It is then transferred to acceptor proteins to initiate the repair complex. Ultimately, PARP-1 undergoes a molecular change that leads to reduced DNA affinity, followed by release from the lesion and reversion to a catalytically inactive state [36, 50, 51].

PARylation is an important process in this mechanism. Negatively charged PARs are covalently bound to the glutamic acid, aspartic acid or lysine residues of the target protein [52]. In this process, PARP uses oxidized NAD+ as a substrate and releases nicotinamide and protons, and the cells consume ATP to restore NAD+ levels. PARylation can produce different effects: it can make the interaction between proteins and DNA unstable or stable, regulate the interaction and function of proteins, promote the activity of target proteins, and cause the proteasome to degrade proteins. Through PARylation, the PARP protein can control a variety of cellular functions, such as DNA replication and transcription, and has important significance in the DNA damage response and cell death [53].

## How PARP inhibitors kill cells with BRCA mutations

PARP inhibitors (PARPis) can bind to the NAD + -binding pocket of PARP-1, produce conformational changes in PARP-1 and stabilize the combination of PARP-1 and DNA. This is referred to as the trapping of DNA − PARP-1 complexes [54]. PARPis bind the catalytic site of PARP-1 and “trap” it at the lesion so that it cannot revert back to an inactive state, and the catalytic cycle is finally broken. This process results in PARP-1 dysfunction. As the cycle stagnation product, PARP-1/DNA nucleoprotein complexes lead to the accumulation of unrepaired SSBs and damage the progression of replication forks (RFs). Ultimately, RF stalling leads to degradation of the highly cytotoxic DSBs [36, 50, 51]. Thus, it follows that PARPis could cause profound damage to SSB repair, while DSB repair plays a vital role in maintaining the integrity of genetic material, which in turn uses HRR as the optimum compensation pathway. Therefore, we can infer that tumor cells will not be able to repair DSBs in the case of HRR deficiency; moreover, under the action of PARPis, the defective cells eventually succumb to synthetic lethality.

There are different forms of synthetic lethality action models for PARPis. As mentioned above and despite the most classic mechanism of BER inhibition and PARPis, synthetic lethality was reported to be related to NHEJ inhibition; HRR defects resulting in HRR-deficient cells depend on NHEJ as a compensate repair pathway so that NHEJ inhibition by PARPis drives cell death in this context [55]. In contrast, another form of synthetic lethality related to NHEJ activation exists. PARP-1 suppresses NHEJ by PARylating Ku70/Ku80 and the catalytic subunit of DNA-PKcs. PARPis cut off this suppression and enhance the error-prone pathways, ultimately leading to increased mutations and cell death [56]. In general, it is still unclear the degree to which these different forms affect the anticancer activity of PARPis. Based on the DNA repair biology mentioned above, the identification of patient subsets with HRR gene mutations and the use of drugs targeting PARP may lead to a new direction for cancer treatment.

In addition to playing an important role in DNA repair, PARP-1 is involved in other biological processes, such as chromatin remodeling, transcriptional regulation, hypoxic response, angiogenesis, epithelial-mesenchymal transition (EMT), and cancer meta-stasis. Most of these processes are related to tumorigenesis and tumor progression and may partly broaden our understanding of the mechanisms of action of PARPis [57].

**Fig. 2:**
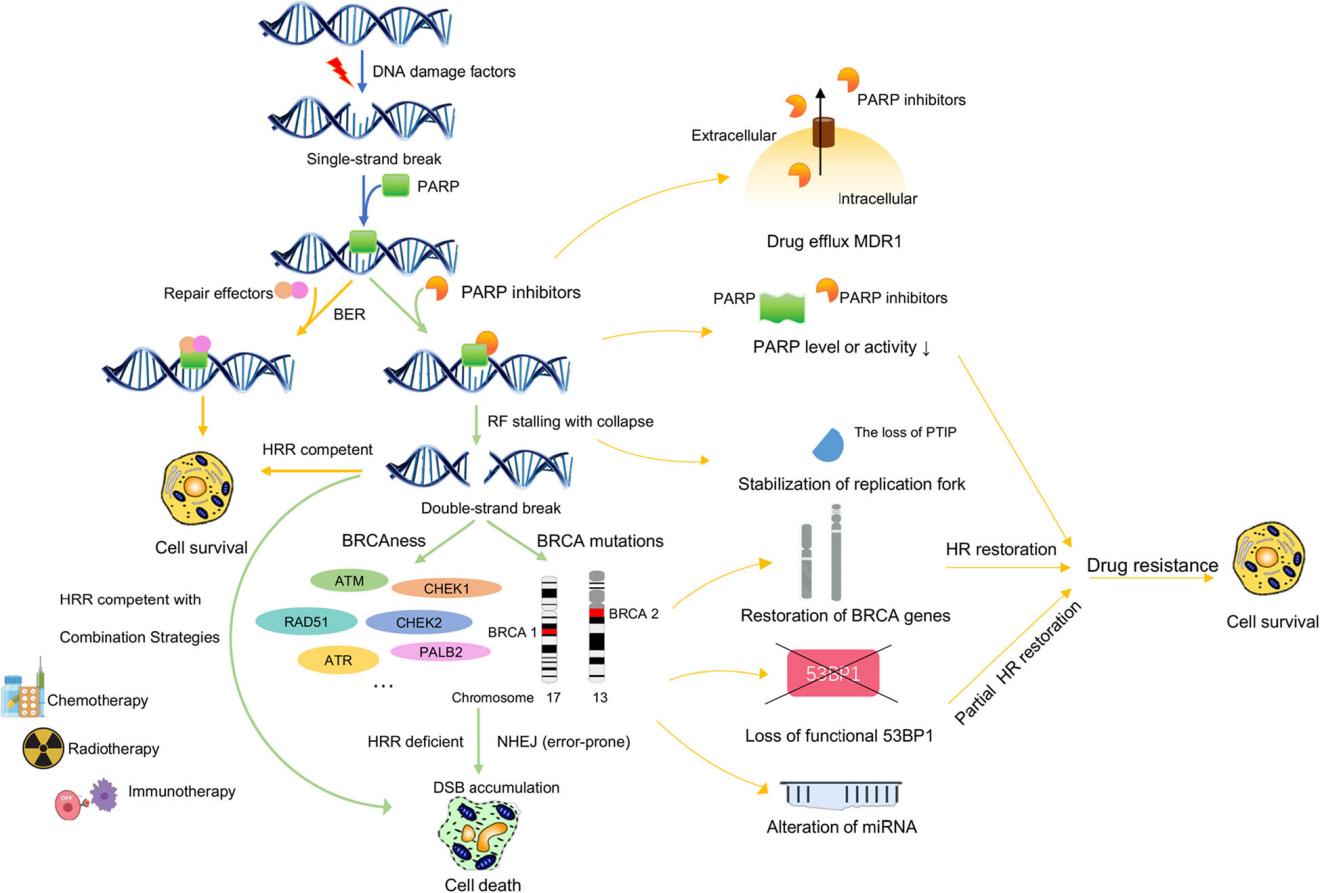
[The action and resistance mechanism of PARP inhibitors] The left panel and right panel of the figure show the action and resistance mechanisms of PARP inhibitors, respectively. The yellow lines and green lines show the pathways leading to cell survival and cell death, respectively

The loss of the wild-type BRCA allele, which is considered a classical tumor suppressor, increases the risks of breast cancer, ovarian cancer, and pancreatic cancer, among others [58, 59]. PARP-1 induces HRR by PARylating BRCA1-associated RING domain protein 1 (BARD1) to promote BRCA1 recruitment to lesions. In the repair process, DSB ends are resected to yield 3′ single-stranded DNA tails; then, they bind to the recombinase protein RAD51 and find a homologous duplex target to form a DNA protein complex structure, namely, a D-loop. The formation of this structure is an important link in DSB repair by homologous recombination [60]. BER is a primary back-up system for HR loss in response to BRCA mutations [37]. However, BRCA1/2 mutant cells cannot undergo DSB repair through HRR, resulting in genomic instability and cell death (Fig. 2).

The model mentioned above is the current mainstream hypothesis. Of course, there are some studies that question this model. Alkylating [61] agent dimethyl sulfate (DMS)-induced SSBs did not accumulate in PARP-1 siRNA-treated cells, demonstrating that PARP-1 is not a BER protein and does not require BER for completion [62]. In addition, it is universally recognized that PARP inhibition delays the induction of SSB repair; however, the steady-state level of SSBs has not been observed to increase in wild-type or BRCA2-defective cells treated with PARPis, so it was concluded that SSBs do not accumulate as a primary lesion after PARP inhibition [63, 64]. Additionally, there is literature indicating that DNA − PARP trapping by PARP-1 inhibitors is not an allosteric effect; rather, it is correlated linearly with catalytic inhibition in biochemical systems and nonlinearly in cells. DSB levels are better related to cell death than trapping [65]. Considering that these relevant issues and others are constantly raised, the mechanism of how PARPis kill BRCA mutant cells still needs further research.

**Fig. 3:**
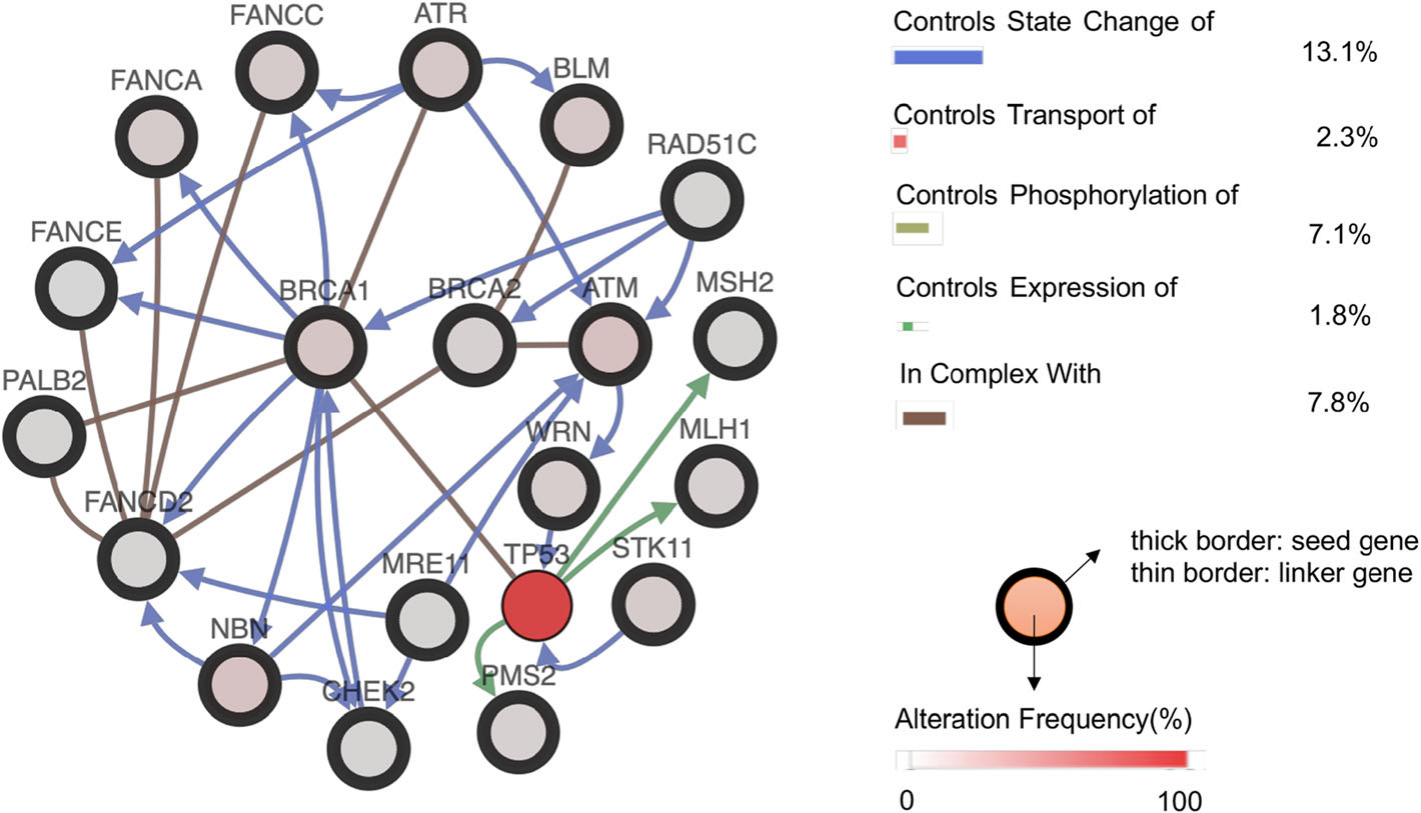
[Gene interactions between BRCA1, BRCA2 and other genes] This gene interaction network is based on the cBioportal website, which shows the different interaction types among BRCA1, BRCA2 and other genes, with neighbors filtered by alterations at 61.7%

At present, the BRCA gene has attracted the most attention among all HRR defect genes, including ATM, ATR, CHEK1, CHEK2, PALB2, RAD51, and the FANC gene family. The term “BRCAness” describes BRCA1 or BRCA2 mutation phenocopies, which represent the situation in which a tumor cell has an HRR obstruction with a germline BRCA1 or BRCA2 deficiency [66, 67]. Theoretically, cancer cells possessing the “BRCAness” phenotype gene defect may be examined for PARPi effectiveness. In addition, upstream molecular mutations regard the “BRCAness” phenotype as a major regulator or critical link; for example, the mutation and deletion of PTEN may regulate RAD51 expression, and PARPi may have good therapeutic effects for those cancer patients [68, 69]. The following gene interaction network (Fig. 3) is based on the cBioportal website and shows the different interaction types among BRCA1, BRCA2 and other genes, which could reveal potential targets for new drugs and provide inspiration for novel ways to decrease drug resistance.

## PARPi resistance

Although PARPis have shown promise in monotherapy as well as combination therapy regimens in clinical trials for several cancers, as with other targeted therapies, the benefits of PARPis have been counteracted by the appearance of resistance (Fig. 2). Therefore, it is essential to understand the resistance mechanisms to achieve curative effects as well as to broaden our basic knowledge regarding the mechanism of action of PARPis.

Because the mechanism of action of PARPis is related to HR deficiency, any methods that restore HR could lead to PARPi resistance in tumor cells. a. BRCA reverse mutations The first identified and most widely accepted pathway of resistance is reverse mutations in BRCA1/2 [70], which was predicted for PARPis as well as platinum-based therapies [71] and is possibly associated with genomic instability [72]. Therefore, targeted deep sequencing of the BRCA mutational profile could predict the drug response to PARPis in recurrent tumors [73]. These reverse mutations often reveal a microhomology signature [74], which demonstrates the outcome of DSB repair through selective error-prone mechanisms in initial HR-deficient cells. Demethylation of the hypermethylated promoter of BRCA1 is another pathway for restoration that has been identified in patient-derived xenograft (PDX) models of tumors with hypermethylation at diagnosis [74]. b. miRNA environment One study has revealed that miR-622 alterations could modulate the NHEJ components to promote PARPi resistance in ovarian cancer cells [75]. In addition, miR-182 downregulation could desensitize BRCA1-proficient breast cancer cells to PARPis [70]. These findings suggest that microRNA expression should be examined to evaluate the PARP drug response. c. Loss of 53BP1 function p53-binding protein 1 (53BP1) is a nuclear protein [76] that plays a key role in striking a balance between HR and NHEJ; NHEJ is promoted by inhibiting the extensive DNA end-resection in HRR [77, 78]. The loss of 53BP1 could reverse the HR defect in BRCA1-deficient cells but not in BRCA2-deficient cells [79]. It is assumed that when resection inhibition by 53BP1 is lost, HR can be reinitiated in a BRCA1-independent manner. This partial restoration of HR explains PARPi resistance in BRCA1 and TP53BP1 double-knockout cells. In subsequent research, RIF1 and REV7 were found to be downstream factors of 53BP1 in NHEJ. Hence, the loss of involved factors would induce PARPi resistance [80]. d. Increases in RAD51 RAD51 is a key HR protein. For example, PARPi-resistant clones were all > 1000-fold resistant to PARPis and possessed the capacity to establish damage-induced RAD51 nuclear foci compared with parental cells, which revealed HR pathway restoration [81]. PALB2–BRCA2 signaling still plays an indispensable role in this HR restoration. Furthermore, cells with restored RF protection depend on RAD51 recruitment for suitable protection. Both PALB2–BRCA2 recruitment to DNA breaks and RAD51 recruitment to stalled forks are ATR-dependent [82]. Therefore, combining PARPis with ATR inhibitors has great potential for decreasing PARPi resistance in tumors with restored HR or restored fork protection [83].

Some non-HR restoration pathways also play a role in the resistance mechanism. RF stabilization One report indicated that the loss of Pax2 transactivation domain-interacting protein (PTIP), a type of HR repair protein, could stabilize and protect RFs and ultimately aid in PARPi resistance [84]. Another factor implicated in replication stress is SLFN11. This protein is not directly associated with RF stability; however, it prolongs S-phase arrest in replication stress by regulating irreversible, prolonged RF stalling [85]. Decreases in PARP Because the target of PARPis is mainly the PARP-1 protein, which is captured on SSBs and cannot be activated, decreases in PARP-1 levels will inevitably lead to PARPi resistance. Thus, PARPi sensitivity would be affected in tumor cells during different stages of tumor development [86]. At the same time, the catalytic activity of PARP itself is also related to the sensitivity of HR-deficient cells to PARPis [64]. PARP protein-catalyzed PARylation is a transient and reversible protein modification in which a PAR chain is added covalently. PARP-1 is mainly responsible for DNA damage in cell PARylation [80]. The cause of PAR chain degradation can be attributed to the activity of PAR sugar hydrolase (PARG) that reverses PARylation. In this manner, PARG acts similarly to PARPis by preventing PAR accumulation. According to a genetic screen of a murine BRCA2-deficient cell line, the absence of PARG was found to be responsible for PARPi resistance [81]. Loss of PARG partially restores PARylation in PARPi-treated cells, which reduces PARP-1 capture on DNA and partially rescues PARP-1-dependent DNA damage signals. Restored PARP-1 catalytic activity prevents uncontrolled RF progression and is sufficient to recruit downstream repair factors, thus leading to PARPi resistance. Decreases in intracellular PARPi levels Pharmacological effects are also associated with the resistance mechanisms. Increased expression of ATP-binding cassette transporters, such as the P-glycoprotein efflux pump (also called multi-drug resistance protein 1 (MDR1)) [87], is relevant to efficient PARPi transport out of tumor cells and thus contributes to drug resistance [88]. The overexpression of drug-efflux transporter genes (Abcb1a and Abcb1b, encoding for MDR1/P-gp, and Abcg2) occurs in most tumor cells [89]. High expression of Abcb1a/b can be observed, especially with high rates of PARPi resistance, in mouse models of mesenchymal carcinosarcomas characterized by epithelial-to-mesenchymal transition phenotypes. ABCB1 overexpression has also been identified in a PARPi-resistant human ovarian cancer cell line, and this resistance could be reversed by cotreatment with the MDR1 inhibitors verapamil and elacridar [90].

## Biomarkers for prognosis and treatment response

In addition to the great possibility of extending PARPi use to pancreatic cancer, it is important to identify suitable candidates among the patient population for the use of this drug and determine how to overcome drug resistance. We should consider the intricate interrelationships between various genes and proteins in the underlying mechanism and develop a more accurate predictive marker. This includes identifying people who are suitable for drug treatment as well as predicting the efficacy of the drug in individual patients, tracking tumor progression during treatment to adjust the dosing as needed, and so on.

Different biomarkers, including BRCA mutations or other genetic mutations associated with HR, have been explored; however, there are still no gold standard methods for identifying patients who are suitable for PARPi treatment. This section will describe some methods for determining prognosis and treatment response.

### BRACAnalysis CDx

BRACAnalysis CDx can detect the occurrence of BRCA mutations in blood samples and is a currently approved molecular companion diagnostic test. However, although it aims to detect BRCA mutations, which are the most reliable and feasible biomarkers used to select applicable patients for PARPi treatment [91], it is insufficient for predicting the involved biomarker curative effects because these mutations are not the only biomarkers involved [92].

### Functional assays for detecting HR defects

Development of functional assays that can detect HR defects to provide alternative methods for identifying BRCAness. a. RAD51 BRCA1/2 or other HR factor-deficient cells cannot form RAD51 nuclear foci efficiently after DNA damage; consequently, RAD51 localization to defined foci in the nuclei, which is one of the specific cellular hallmarks of HR dysfunction [93] and can predict chemotherapy effects [94], can be identified through immunofluorescence microscopy. Overexpression of the mitotic serine/threonine kinase aurora A damages RAD51 recruitment and is thus involved in tumor cell resistance to PARPis [93]. b. H2AX The H2AX histone is also an important HR-associated marker that is phosphorylated to form gH2AX and creates a place for assembling DNA repair and chromatin remodeling factors at DSB foci [95]. This protein can be detected through immunofluorescence using a gH2AX antibody, and it was analyzed in primary ovarian cancer cells by a combination of gH2AX/RAD51 immunofluorescence [94]. c. ATM ATM can phosphorylate the H2AX histone to form γH2AX and act as a cell cycle checkpoint trigger [96]. Deficiency of this series of factors in this pathway, including ATM, checkpoint kinase (CHK) 1, CHK2, and the cyclin B1/cyclin-dependent kinase (CDK) 1 complex, would lead to synthetic lethality upon PARPi use [97]. The MRE11 − RAD50 − Nijmegen breakage syndrome 1 (NBS1) complex (MRN) can activate ATM to induce HRR [98] so that MRE11 disturbance would weaken HRR [99]. d. PI3 kinase (PI3K)/AKT/mTOR pathway bold formatting Aberrations in the PI3K/AKT/mTOR pathway have also been reported to be associated with HRR. PI3K inhibition can decrease BRCA expression, causing PARPi inhibition [82]. Additionally, PTEN is a factor regulating HRR. Although it is not a component of the HRR pathway, it can alter HRR activity as a tumor suppressor to inactivate the PI3K/AKT pathway, and PTEN loss can result in HRR deficiency [69]. e. Fanconi anemia (FA) proteins [100] and epigenetic BRCA1 inactivation [101] were also shown to act as potential biomarkers for PARPis.

### PARP-1 related biomarkers

PARP-1 is the main target of PARPis, but there are no pathways for detecting PARP-1 as a genetic biomarker. Instead, factors associated with PARP-1 can serve as predictive markers [102]. For example, CDK5 silencing was reported to be responsible for synthetic lethality with PARP-1 inhibitors. REV7 was shown to be downstream of 53BP1, which induces the DSB repair pathway in BRCA mutant cells, and the loss of 53BP1 or REV7 confers PARPi resistance [103]. Fused erythroblast transformation-specific (ETS) genes and the expression status of PARP-1 and forkhead box O (FOXO) 3A have also been related to OS and RFS in gastric cancer [104].

### Error-prone NHEJ pathway-related biomarkers

According to the Foundation Medicine LOH assay (Foundation Medicine, Inc., Cambridge, MA; in collaboration with Clovis Oncology, Inc., Boulder, CO), HR-deficient tumors rely on the error-prone NHEJ pathway for repair and can undergo large-scale LOH.

In this method, DNA is extracted from formalin-fixed paraffin-embedded (FFPE) tissue, and next-generation sequencing is then performed. LOH scores can be determined by assessing more than 3500 single nucleotide polymorphisms (SNPs) and sequencing coverage. This analysis can also distinguish germline mutations from somatic BRCA1/2 mutations. However, it cannot reflect the loss of functional HR as accurately as the BRCA1/2 mutant status [105]. Another biomarker technique in the pipeline is Myriad’s HRD assay (Myriad Genetics, Salt Lake City, UT). This is a combination of three scores using DNA extracted from FFPE samples [106]. These two approaches do not take into account possible reverse mutations of BRCA1/2; therefore, inaccurate marker detection caused by BRCA recovery is also avoided.

## Clinical trials related to pancreatic cancer

As discussed above, PARPis can sensitize cancer cells to DNA-damaging chemotherapies. Supported by this rationale, several clinical trials have focused on developing clinically useful PARPi drugs—both single agents and combination therapies—for treating pancreatic cancer. Clinical trials of several PARPi drugs are currently underway, and thus far, olaparib, rucaparib and niraparib are commercially available in the US or Europe [107]. The most advanced clinical application of PARPi drugs is ovarian cancer. We predict that PARPis will have broad application prospects in other malignant tumors with BRCA mutations. Additionally, these clinical results will further promote research into the underlying mechanism of PARP, and a better understanding of this mechanism will further guide the development of compatible clinical drugs and reduce subsequent drug resistance.

At present, there are almost 26 registered PARPi agents (Table 1). A search for “pancreatic” yielded 31 relevant clinical trial records on the http://clinicaltrails.gov website. Among them, there are 10 records for olaparib; the monotherapy trials are mostly in phase II or III, while the combination therapy trials are mostly in phase I or II. There are 9 records for veliparib, and the combination therapy trials are mostly in phase I or II. In addition, rucaparib, talazoparib and niraparib have 4, 3, and 3 records, mostly in phase I or II.

**Table 1:** Clinical trials of PARP inhibitor drugs for pancreatic cancer

Trial ID	Therapeutic Drugs	Phase	Status	Treatment Setting	Primary Outcomes
NCT02677038	Olaparib	II	Recruiting	Metastatic PAC Patients must be germline BRCA 1 or 2 negative	Objective tumor response rate
NCT02511223	Olaparib	II	Unknown	Metastatic PAC with BRCA 1/2 mutations negative but loss of ATM	Objective response rate
NCT01078662	Olaparib	II	Active, not recruiting	Advanced tumors with BRCA1/2 mutation, including pancreatic cancer	Tumor response rate
NCT02184195	Olaparib Placebo	III	Active, not recruiting	Metastatic adenocarcinoma of the pancreas with germline BRCA1/2 mutations	Progression-free survival
NCT01296763	Olaparib Irinotecan Cisplatin Mitomycin-C	I	Completed	Advanced pancreatic cancer	Maximum-tolerated dose
NCT00515866	KU-0059436 (AZD2281) Gemcitabine	I	Completed	Advanced or metastatic unresectable PAC	Maximum-tolerated dose or tolerable and effective dose
NCT03682289	Olaparib ATR Kinase Inhibitor AZD6738	II	Recruiting	Locally advanced or metastatic solid tumor malignancy, including pancreatic cancer	Objective response rate
NCT03851614	Olaparib Cediranib	II	Recruiting	Mismatch repair-proficient colorectal cancer Pancreatic adenocarcinoma Leiomyosarcoma	Genomic and immune biomarkers
NCT02498613	Olaparib Cediranib Maleate	II	Recruiting	Metastatic or unresectable malignancy, including PDAC	Objective response rate
NCT03878524	SMMART Therapy Including Olaparib	I	Not yet recruiting	Breast cancer Prostate cancer Pancreatic cancer Acute myelogenous leukemia	The number of participants to complete first dose of first SMMART therapy
NCT00892736	Veliparib	I	Completed	Solid tumors with BRCA1/2 mutations, including pancreatic cancer	Maximum-tolerated dose Dose-limiting toxicities Recommended phase II dose
NCT01908478	Veliparib Gemcitabine	I	Active, not recruiting	Pancreatic cancer	Maximum-tolerated dose
NCT01489865	ABT-888 mFOLFOX-6	I and II	Active, not recruiting	Metastatic pancreatic cancer	Dose-limiting toxicities
NCT02890355	Veliparib Fluorouracil Irinotecan Hydrochloride Leucovorin Calcium	II	Active, not recruiting	Metastatic pancreatic adenocarcinoma, recurrent pancreatic carcinoma, stage IV pancreatic cancer	Overall survival
NCT01585805	Veliparib Cisplatin Gemcitabine Gemcitabine Hydrochloride	II	Active, not recruiting	Locally advanced or metastatic pancreas adenocarcinoma with a BRCA1/2 or PALB2 mutation	Optimal dose Response rate
NCT01282333	Veliparib Cisplatin Gemcitabine Hydrochloride	I	Terminated	Advanced biliary/pancreatic cancer, urothelial cancer, non-small cell lung cancer	Maximum-tolerated dose
NCT02831179	Veliparib Capecitabine Temozolomide	I	Withdrawn	Metastatic unresectable neuroendocrine tumors, non-functional pancreatic neuroendocrine tumors, pancreatic glucagonoma, pancreatic insulinoma	Maximum-tolerated dose
NCT01233505	Veliparib Capecitabine Oxaliplatin	I	Terminated	BRCA-related solid tumors, including pancreatic cancer	Dose-limiting toxicities Maximum-tolerated dose
NCT00576654	Veliparib Irinotecan Hydrochloride	I	Active, not recruiting	Malignant solid neoplasms, including pancreatic cancer	Optimal biologic dose
NCT03140670	Rucaparib	II	Recruiting	Locally advanced or metastatic pancreatic cancer	Number of adverse events
NCT02042378	Rucaparib	II	Completed	Pancreatic cancer, pancreatic ductal adenocarcinoma	Overall response rate
NCT03337087	Rucaparib Fluorouracil Leucovorin Calcium Liposomal Irinotecan	I and II	Recruiting	Pancreatic, colorectal, gastroesophageal or biliary adenocarcinoma	Maximum-tolerated dose
NCT02711137	Rucaparib INCB057643 Gemcitabine Paclitaxel Abiraterone Ruxolitinib Azacitidine	I and II	Terminated	Solid tumors, including pancreatic cancer	Safety and tolerability
NCT01286987	Talazoparib	I	Completed	Locally advanced or metastatic solid tumors, including pancreatic cancer	Number of participants with an objective response
NCT02567396	Talazoparib	I	Withdrawn	Metastatic or unresectable malignancies including pancreatic adenocarcinoma	Incidence of toxicity Recommended phase 2 dose Tolerability
NCT03637491	Talazoparib Avelumab Binimetinib	II	Recruiting	Locally advanced or metastatic solid tumors, pancreatic cancer	Dose-limiting toxicity
NCT03601923	Niraparib	II	Recruiting	Pancreatic cancer	Progression-free survival
NCT03553004	Niraparib	II	Recruiting	Pancreatic cancer	Objective response rate
NCT03404960	Niraparib + Nivolumab Niraparib + Ipilimumab	I and II	Recruiting	Pancreatic adenocarcinoma	Progression-free survival
NCT02244489	Momelotinib Capecitabine Oxaliplatin	I	Terminated	Relapsed/refractory metastatic pancreatic ductal adenocarcinoma	Incidence of dose-limiting toxicities Safety
NCT02101021	Momelotinib Placebo to match Momelotinib Nab-paclitaxel Gemcitabine	III	Terminated	Metastatic pancreatic ductal adenocarcinoma	Dose-limiting toxicity Overall survival

The following section describes the different PARP drugs according to the preliminary data reported from the clinical trials.

### Olaparib

Olaparib (Lynparza™) is an oral PARPi that was recently approved for the treatment of advanced ovarian cancer, and it remains the only agent approved to date [108].

According to a prospective, multicenter, nonrandomized phase II study using olaparib monotherapy for patients with a germline *BRCA1/2* mutation and recurrent cancer, including pancreatic cancer with prior gemcitabine treatment, olaparib (capsule formulation) was administered at a dose of 400 mg twice per day. As the primary efficacy end point, the tumor response rate was 21.7%, and stable disease ≥8 weeks was observed in 35% of patients with pancreatic cancer. In the first-line setting, the disease response rate for gemcitabine plus nab-paclitaxel was 23%, and that for FOLFIRINOX (leucovorin, fluorouracil, irinotecan, and oxaliplatin) was 31.6%. In the second-line setting, the response rates to chemotherapy were generally < 20%. Olaparib was the third-line therapy in this study, and the results may support its further use in metastatic pancreatic cancer [109].

The newly reported outcomes of the POLO (Pancreatic Cancer Olaparib Ongoing) trial (NCT02184195) for patients with metastatic pancreatic cancer that had not progressed during platinum-based chemotherapy and a BRCA1 or BRCA2 mutation have indicated that olaparib can be used for maintenance therapy for pancreatic cancer. In this double-blind, placebo-controlled, phase III trial, an intervention was assigned randomly to 154 patients (92 received olaparib, and 62 received placebo). Olaparib or placebo was administered at a dose of 300 mg twice daily, and median progression-free survival was then evaluated. The results show that the olaparib group had prolonged survival compared to the placebo group (7.4 months vs. 3.8 months) [110].

Regarding combination therapy with olaparib, a phase I study (NCT00515866) was completed that aimed to determine the safety, tolerability, and maximal tolerable dose (MTD) of olaparib combined with gemcitabine in patients with advanced solid tumors. Olaparib combined with chemotherapeutic agents was found to exhibit increased hematological toxicity according to previous studies. A combination of olaparib 100 mg BID (capsule formulation; intermittent dosing on days 1–14) with gemcitabine 600 mg/m2 was administered i.v. on days 1, 8, and 15 every 4 weeks to 66 advanced solid tumors patients in a randomized dose-expansion trial; according to adverse event (increased alanine aminotransferase levels, neutropenia, and febrile neutropenia) observation, this regimen had an acceptable tolerability profile, and this dose combination could be used in further studies [111].

Another phase I study (NCT01296763) of olaparib combination therapy was performed to determine the MTD of olaparib in combination with irinotecan (olaparib + IC) as well as the safety and tolerability of adding mitomycin (olaparib + ICM). The trial results revealed that olaparib in combination therapy showed significant toxicity in PDAC patients with IC or ICM. Moreover, the results of this trial did not show an acceptable risk/benefit profile to support further study [111].

### Veliparib

In a single-arm phase I clinical trial (NCT01908478) of gemcitabine, radiotherapy and dose-escalated veliparib in locally advanced pancreatic cancer (LAPC) patients, weekly gemcitabine treatment with daily IMRT and dose-escalated veliparib was assigned to 30 patients diagnosed with naïve LA or borderline resectable pancreatic cancer. The primary MTD endpoint for veliparib was 40 mg BID with 400 mg/m2 gemcitabine and RT (36 Gy/15 fractions). This study confirmed that veliparib is safe and well tolerated in combination therapy with gemcitabine and RT for patients with LAPC [112].

### Rucaparib

Rucaparib is also an oral PARPi. A phase 2 study (NCT02042378) focused on the efficacy and safety of rucaparib in BRCA1/2 mutant patients with measurable locally advanced/metastatic pancreatic cancer. Nineteen subjects (sixteen had germline mutations, and three had somatic mutations) received oral rucaparib (600 mg twice daily) after the administration of one to two prior chemotherapy regimens. Two partial responses and one complete response (CR) were confirmed (objective response rate, 15.8%; 3 of 19). The disease control rate (CR, partial response, or stable disease for ≥12 weeks) was 31.6% (6 of 19) for all patients. Grade ≥ 3 adverse events included anemia (31.6%), fatigue (15.8%), and ascites (15.8%). This study provided evidence to show that rucaparib has an acceptable safety profile and is beneficial in advanced pancreatic cancer patients [113].

### Talazoparib

Talazoparib (MDV3800 or BMN 673) is another type of novel and selective PARPi that is more potent than earlier-generation PARP-1/2 inhibitors. A two-part, multicenter, dose-escalation, phase I study (NCT01286987) was completed to demonstrate the antitumor activity and MTD of talazoparib. Four of the 13 patients with pancreatic cancer showed clinical benefit (CBR, 31% ≥16 weeks). The MTD of talazoparib was 1.0 mg/day, and it was well tolerated overall with good oral bioavailability and rapid absorption [114].

## The Prospect of PARPi use in pancreatic cancer

In summary, we found that there are quite a few mechanisms that have been pursued in the exploration of PARPis, as well as corresponding resistance studies. A number of clinical trials for PARPis in pancreatic cancer are underway and have achieved some interesting results. The most widely investigated mechanism of action is the generation of DNA damage that cannot be effectively repaired in BRCA gene-deficient cells according to the synthetic lethality principle. However, there are a number of genes in addition to BRCA that possess “BRCAness” that could elicit HRR defects. Some of these genes are also found in certain proportions in mutated gene sequencing studies in pancreatic cancer, which undoubtedly extends the targeting sites of PARPis for this disease. Moreover, PARPis have been shown to induce sensitivity to chemotherapy and radiation. Platinum-based chemotherapy destroys the ability to repair double-stranded DNA, which means that platinum-sensitive tumor cells are likely to have defects in HRR, and platinum-based chemotherapy acts coordinately with PARPis. However, the mechanisms in this process have not yet been fully elucidated.

The only accepted PARPi for clinical application in pancreatic cancer is olaparib. According to the POLO trial, it has been used as a monotherapy for maintenance treatment in patients with metastatic pancreatic cancer who do not exhibit disease progression for > 16 weeks after first-line, platinum-based chemotherapy. The POLO trial opens the door to a new era of precision treatment according to molecular markers and phase III clinical research for pancreatic cancer maintenance treatment. The results will undoubtedly promote the development of genetic testing for pancreatic cancer. There are still some issues regarding the POLO trial that are worthy of discussion. First, the POLO trial results showed improvements in only PFS, and there were no significant differences in OS. Such a modest achievement is insufficient to warrant the implementation of a drug for widespread use in a certain cancer, so the clinical trial sample sizes should be increased. Further investigations are urgently needed to determine the broad applicability of this drug. Second, whether PARPi can be used as a first-line treatment, preoperative neoadjuvant therapy or adjuvant therapy in addition to maintenance therapy requires further study, and whether it can be used in combination with other chemotherapy, radiotherapy or targeted drugs to achieve better efficacy is also worthy of investigation. Moreover, the POLO study has limited benefits; the proportion of patients with BRCA mutations is small among patients with pancreatic cancer, so it is particularly important to extend the application to patients without BRCA mutations.

According to the POLO3 study, the adverse reactions to olaparib are generally controllable [110], and the adverse events are similar to those experienced with other types of tumors. The results show that among the patients, 35.2% discontinued treatment, 16.5% reduced the drug dose, and 5.5% completely terminated treatment, which suggests that toxicity management is necessary for PARPi application in pancreatic cancer patients. There is still a need to be cautious of other malignant adverse events reported for other studies and other PARPi agents that may impede clinical applications. According to some clinical reports, some patients with confirmed BRCA mutations responded poorly to PARPi therapy [115], while other ovarian cancer patients without significant BRCA deficiency responded well. This result suggests that it is imprudent to assume that patients with BRCA mutations will be sensitive to PARPis.

According to PARPi clinical trials for ovarian cancer, approximately 80% of patients with highly serous ovarian cancer do not have BRCA mutations; thus, extending the benefits of PARPi first-line maintenance therapy from patients with BRCA mutations to patients with HRD can greatly overcome these limitations. The current PAOLA-1 [116], PRIMA [117], and VELIA [118] studies include patients with no BRCA mutation, and their respective PARPi regimens have resulted in a better PFS. This indicates that the population with a survival benefit due to PARPi first-line maintenance therapy can be expanded. However, the results of most current clinical studies for various cancer types indicate significant prolonging of PFS; although OS data are not yet mature, there is a benefit trend but no significant prolongation. The limitations of PARPi in the application to other cancer types should also be considered in its application to pancreatic cancer.

With regard to biomarkers for prediction and diagnostic efficacy, the best predictor of drug response remains uncertain. Most clinical trials recruit patients based on pathological subtypes (such as TNBC and HGOSC) or have used BRCA mutation analysis (germline and/or somatic cells) as part of the selection criteria, but few biomarker tests (such as other HR gene defects) were used for its inclusion requirements. Today, some clinical trials have used biomarker analysis as part of their outcome indicators, and these future research results can help to identify which biomarkers are suitable for inclusion in subsequent experiments. BRCA is not sufficient for use as the best candidate biomarker for evaluating PARPi response, and a list of related markers needs to be further validated in the future. In addition, there is currently no clear evidence explaining why the PARPi response exists in tumors that do not have typical HR repair gene mutations. As mentioned earlier, the PARP protein has a mechanism of action beyond DNA repair, so the benefits of PARPis may not be limited to BRCA or even BRCAness-related tumors. Therefore, more research on the molecular mechanism of PARPis and more clinical trials on the extensive application of PARPis in pancreatic cancer will be critical to advance the field of PARP inhibition therapy and to improve patient selection and subsequent clinical outcomes.

## Data Availability

The materials that support the conclusion of this review have been included within the article.

## Abbreviations

BER: Base excision repair
BRCA: Breast cancer susceptibility genes
DMS: Dimethyl sulfate
DSB: Double-stranded DNA break
HRR: Homologous recombination repair
MDR1: Multi-drug resistance protein 1
MMR: Mismatch repair
NAD: Nicotinamide adenine dinucleotide
NER: Nucleotide excision repair
NHEJ: Nonhomologous end-jointing
PAR: Poly (ADP-ribose)
PARP: Poly (ADP-ribose) polymerase
PARPi: Poly (ADP-ribose) polymerase inhibitor
PFS: Progression-free survival
POLO: Pancreatic Cancer Olaparib Ongoing
PTIP: Pax2 transactivation domain-interacting protein
RF: Replication fork
SDL: Synthetic dosage lethality
SSB: Single-stranded DNA break
XRCC1: X-ray repair complementing defective repair in Chinese hamster cells 1
